# Evaluating the use of role-play simulations in teaching management of medical emergencies in the dental clinic

**DOI:** 10.1186/s12909-023-04818-9

**Published:** 2023-11-03

**Authors:** Maisa O. Al-Sebaei

**Affiliations:** Department of Oral and Maxillofacial Surgery, Faculty of Dentistry, King Abdulaziz University, 21589 Jeddah, PO box 80209, Saudi Arabia

**Keywords:** Emergencies, Simulation, Role-play, Dentistry

## Abstract

**Background:**

Practical hands-on training is necessary for clinical competence in managing medical emergencies in the dental setting. Therefore, the King Abdulaziz University Faculty of Dentistry developed a role-play simulation-based clinical module for training clinical years (Years 4, 5, and 6) dental students in medical emergency management between 2016 and 2020. This study aimed to assess the knowledge and skills of years 4, 5, and 6 dental students before and after the completion of the role-play simulation-based medical emergency training module.

**Methods:**

A mandatory four-hour annual training module was designed consisting of a 45-minute lecture on the medical emergency basic action plan and overview, four hands-on stations, and six role-play simulation-based medical emergency stations. A 16-item multiple choice test was used to assess the knowledge of the students before and after the training module every academic year. An objective structured clinical exam (OSCE) on a medical emergency scenario was administered to the students graduating each year. The difference between the pre- and post-module test scores and the one-time OSCE pass/fail rate were analyzed statistically using appropriate tests.

**Results:**

A total of 846 students participated in the study between 2017 and 2020: 275 once, 483 twice, 87 thrice, and one participated four times; in total, 1,506 assessments were conducted. Overall, the pre-test and post-test mean scores were 9.4 ± 3.0 and 13.5 ± 1.6, respectively. All students showed significant improvements in the post-test scores compared to the pre-test scores. Year 4 students showed the highest improvement, followed by Year 5 and Year 6. There was a statistically significant association between the pass/fail rate of the OSCE station and the number of times the module was taken.

**Conclusions:**

The significant improvement in the scores of clinical year students in this study supports an annual mandate for all dental students to undergo simulation-based competency training in their clinical years. Teaching institutions are recommended to utilize simulation and hands-on instruction to teach medical emergency management.

## Background

Medical emergencies can occur before, during, or after dental treatment. They can occur secondary to stress and fear of the dental procedure or can be directly related to underlying co-morbidities [1–3]. They can range from a vasovagal syncope to a more serious medical event, such as bronchospasm or anaphylaxis [4, 5]. Therefore, the dental clinic must be adequately prepared with proper equipment, medication, training, and mock drills [6–8]. Medical emergencies at a dental office occur at an annual frequency of 0.5 to 2 events per dentist [6, 7], with nearly 50–70% of all dental practitioners experiencing one or more medical emergency events annually [3]; In a study on Saudi Arabian dentists, 67% of respondents reported experiencing a medical emergency [9]. In academic dental institutions, the dental clinics of students are more likely to encounter medical emergencies; in a study from Australia, most (72%) of medical emergencies were recorded at the clinics of dental students [2]. Moreover, dental students are more likely to be underprepared and underconfident in managing medical emergencies arising in dental clinics [10, 11]. Therefore, it is necessary to improve the preparedness of dental students in this regard. However, lecture-based methods may not be the best methods when imparting knowledge and skills related to the management of medical emergencies to dental students. The use of non-didactic innovative learning methods has been explored in dental education, especially while imparting skills related to communication and professionalism [12, 13] and when teaching the management of medical emergencies [14, 15]. Therefore, it is crucial that the methodology for teaching dental students about the management of medical emergencies in the dental clinic is not be limited solely to didactic instruction; practical hands-on training is necessary for clinical competence [16, 17]. Role play-based teaching of cardiopulmonary resuscitation to dental students has been shown to significantly improve the students’ theoretical knowledge as well as skills related to cardiopulmonary resuscitation [14]. Similarly, the use of a flipped classroom combined with a micro-class approach has also shown a significantly better performance of dental students regarding knowledge and skills related to cardiopulmonary resuscitation [15]. The significantly superior knowledge and skills of students when using these hands-on methods versus the traditional lecture method in these studies supports a greater use of hands-on methods, such as role-play, in dental students’ training on medical emergency management.

Reports on the management of medical emergencies in dental clinics in Saudi Arabia [9, 17, 18] and in other countries [19–22] have shown a need for better training of dental students in managing medical emergencies. Recognizing this need for preparing dental students to manage medical emergencies, a major part of the oral surgery course curriculum at the King Abdulaziz University, Faculty of Dentistry (KAUFD) has been devoted to teaching the management of medically compromised patients and medical emergencies in the dental office. At KAUFD, department of oral and maxillofacial surgery, a novel hands-on role-play simulation-based medical emergency training module was initiated in the academic year of 2016–2017 and has been since used uninterruptedly. This module, which is mandatory for the students of Years 4, 5, and 6 on an annual basis, aims to ensure patient safety by preparing the dental hospital and training the dental students on the proper preparedness and management of medical emergencies. This study aimed to assess the knowledge and skills of years 4, 5, and 6 dental students before and after the completion of the role-play simulation-based medical emergency training module.

## Methods

This study was conducted at the KAUFD that offers a six-year program leading to the award of a Bachelor of Dental Surgery (BDS) degree. Upon completion of the program, the students are required to complete a compulsory intern year. The KAUFD was established in 1987 and to this date, 30 undergraduate classes have graduated with a BDS degree. Currently, the school has 12 postgraduate programs in various disciplines of dentistry. In the recent years (2020–2023), the total number of enrolled undergraduate and post graduate students ranged from 853 to 1000, while the strength of the graduating class ranged from 151 to 198, and the total number of faculty members ranged from 504 to 568. The University Dental Hospital serves as the primary facility where students receive pre-clinical and clinical training and treat their patients. It consists of three buildings, each with three floors, encompassing a total of 364 dental clinics.

The undergraduate dental students of the clinical years (Years 4, 5, and 6) at the KAUFD were the subjects for this study; all students were enrolled in the study with no exclusions as this program was a mandatory annual requirement for the students of the clinical years (Years 4, 5, and 6) of the BDS program.

### Module design

**Fig. 1:**
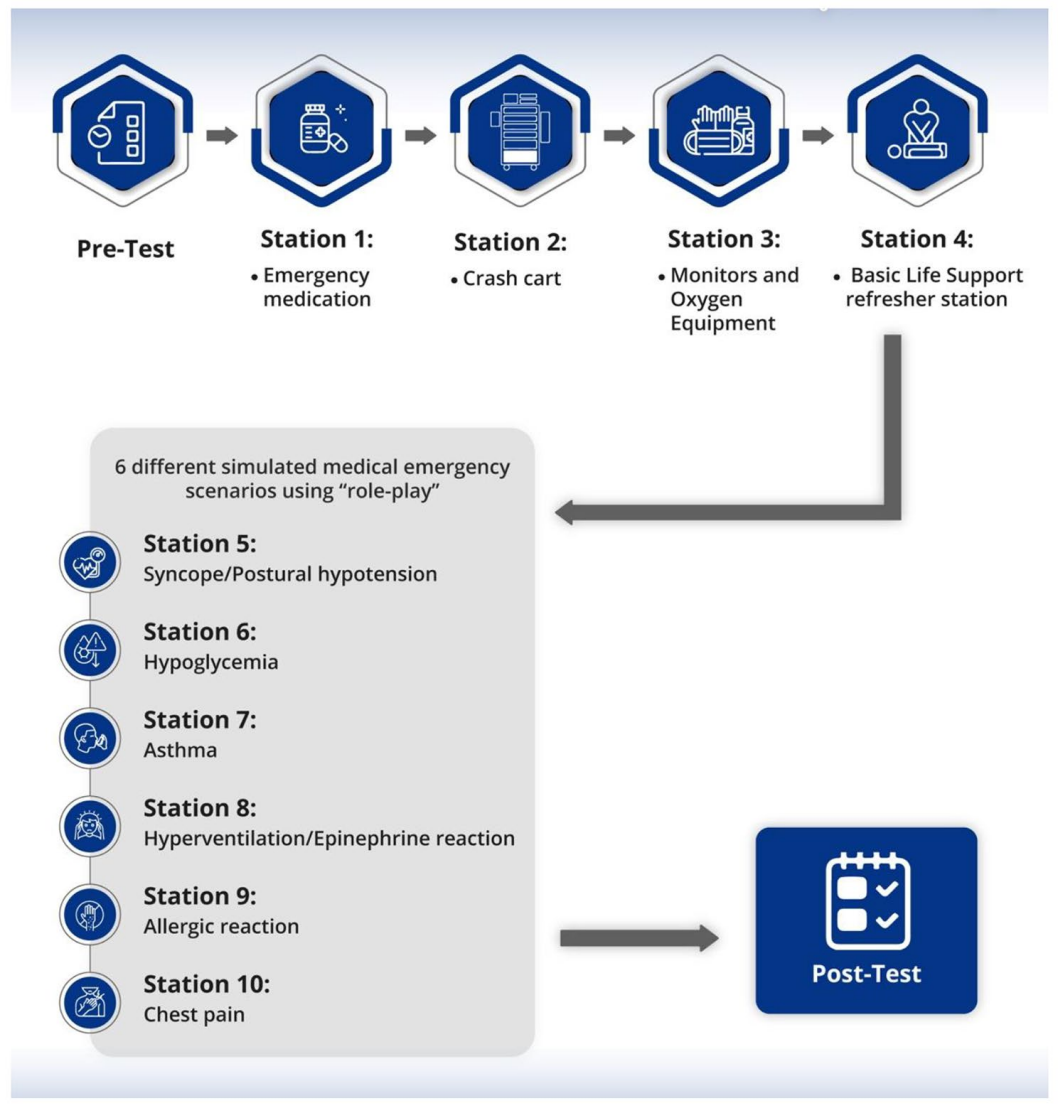
Central illustration for the training module: Four hands-on stations and six-role-play simulation stations

A 4-hour training module was designed that was to be delivered to the students in one day. It consisted of a pre-test, a 45-minute lecture, four hands-on stations, six role-play simulation-based medical emergency stations, and a post-test (Fig. 1).

### Lecture

Through the lecture, the students were expected to learn the essentials of prevention and preparation of medical emergencies in a dental setting; it aimed to develop the aspects of knowledge and behavior in the students. The lecture comprised a comprehensive discussion on the types and prevalence of medical emergencies in the dental clinics, with emphasis on the essential components for effective prevention and preparedness of the healthcare providers and facilities. This included a thorough discussion on medical history and vital signs assessment, conducting mock drills, establishing protocols, ensuring the availability of equipment, assigning tasks, and understanding the basic medical emergency medications. The basic action plan in case of any medical emergency was presented in detail. To enhance the educational experience, the lecture featured two brief videos demonstrating the proper response to two commonly encountered medical emergencies: syncope and hypoglycemia. Additionally, a third video was shown illustrating an improper approach as well as multiple errors in managing the medical emergency, and the students were asked to provide comments, critique to foster critical thinking and problem solving.

### Hands-on stations

In the hands-on stations, the students were supposed to demonstrate a through hands-on approach to the use of emergency drugs, crash carts, monitors, glucometers, and oxygen delivery equipment; it aimed to target the development of necessary skills and behavior. The dental students were divided into small groups, each accompanied with an instructor. Each group underwent 15 min of training at each of the following 4 stations.

Station 1: Emergency medication: The students received a detailed instructor-led explanation and demonstration of the basic emergency medication, including the indications, dose, route, and special considerations.

Station 2: Crash cart contents: The difference between a code blue cart and basic emergency cart was demonstrated by the instructor. The students had the opportunity to explore the contents of the emergency cart and familiarize themselves with the essential items.

Station 3: Monitors and oxygen equipment: The students received an instructor-led demonstration on operating the vital signs monitor, troubleshooting techniques, and the correct application of the blood pressure cuff and pulse oximeter. Further, this station included a demonstration of the oxygen tank and various oxygen delivery devices, such as tank opening procedures, tank fill verification, and proper application of the oxygen delivery devices. Students had the opportunity to practice applying the blood pressure cuff, pulse oximeter, and oxygen facemask on themselves or their classmates.

Station 4: Basic Life Support (BLS) refresher station: The students were given a brief review and demonstration of the American Heart Association BLS protocol. The BLS-certified students demonstrated the one- and two-responder protocols on the BLS mannequin.

### Role-play stations

At the six rotational simulation-based role-play stations, the students were required to practice the management of different medical emergency scenarios using participant simulation based on “role-play”; it targeted the aspects of knowledge, skills, and behavior. These scenarios were carefully selected to align with the most commonly reported medical emergencies in the literature and the latest recommendations [23–27]. The topics covered were syncope/postural hypotension, hypoglycemia, asthma, hyperventilation/epinephrine reaction, allergic reaction, and chest pain.

At each instructor-led, role-play station, each participant was assigned a role, such as that of a patient, operator, assistant 1, or assistant 2, and provided with a badge indicating their assigned role. The specific task for each role was written on the back of the badge. The participant was expected to fully enact the whole scenario until they demonstrated competency, as assessed by checking all items on the provided checklists. The instructor guided the students while adhering to the rubric to ensure all steps were accomplished. Roles were switched between students at the subsequent stations (Fig. 1).

### Outcome measures

Two outcome measures were recorded and analyzed: (a) the score difference (gain) between the pre- and post-module tests and (b) The pass/fail rate in the objective structured clinical exam (OSCE) station on medical emergencies that was conducted among Year-6 students.

*a) Pre- and post-module tests*: A 16-item multiple choice test with four choices and only one correct answer was developed. Content validity and item analysis were used to validate the test. To establish content validity, the concept of the module was clearly defined along with the learning objectives for the theoretical framework and clinical skills. The items in the test were formulated to align with the construct. An expert panel of four experienced faculty members reviewed the test to ensure that it aligned with the learning objectives and covered the relevant content. Furthermore, the test was checked for ambiguity, clarity, and scientific accuracy. A pilot test was conducted on a group of 60 undergraduate students. The test was reviewed once more by the expert panel, where issues such as errors, misleading and confusing items were revised or excluded. This was followed by an item analysis for the pilot test. The following statistics were analyzed; (i) Difficulty index: items below 0.3 and 0.8 were excluded, (ii) Discrimination index: items less than 0.3 were excluded; (iii) Distractor analysis: non-functional and confusing distractors were removed. The answer key and scoring were checked for inconsistencies. Reliability of the test was calculated using Kuder-Richardson Formula 20 (KR-20) coefficient. The test consisting of 16 items yielded a KR-20 value of 0.74 indicating an acceptable level of internal consistency.

The test was administered to the students before the training module (pre-test) and immediately after the course (post-test). The same test was administered in the calendar years 2017–2020. The pre-course and post-course test scores were recorded for all Year 4, 5, and 6 students and were tabulated in a spreadsheet [Excel version 16.66.1, Microsoft Inc., USA].

*b) Objective structured clinical exam*: At the KAUFD, the year-6 OSCE is conducted as an annual exam for the graduating class. It consists of 15 stations that assess various clinical competencies in different dental disciplines. The students need to pass each OSCE station to graduate. The students are allowed to repeat the failed stations until competency is achieved.

In this study, one OSCE station was dedicated to the assessment of the students’ competency in medical emergency management. At the medical emergency OSCE station, the threshold set for passing was set at 70%. Each student was presented with a single simulated medical emergency (syncope or hypoglycemia), performed by a standardized patient (SP). The student needed to demonstrate competency in recognizing and managing that medical emergency.

Validity and reliability of this OSCE station were established as follows. To establish the validity of the OSCE, the intended outcome, specific skill set, and competency intended to be assessed were clearly defined. The student needed to demonstrate the necessary steps for medical emergency prevention (eliciting medical history and vital signs), the ability to recognize the emergency, competency in managing the medical emergency, including proper chair positioning, selection of the appropriate medication if applicable, correct application of the oxygen delivery device, diagnosing the type of medical emergency systematically, whilst demonstrating leadership, teamwork, and an overall systematic approach. The scenarios were developed by the author, which then underwent a rigorous review process by a panel of domain experts to ensure it encompassed the targeted competency, was aligned with the learning outcomes and blueprint, and that the instructions were clear to the SP, student, and examiner, and that there was a well-defined rating and scoring guide in the rubric. The rubric was a 20-item checklist for every step in the case scenario. Critical errors in the rubric were defined as steps missed or incorrectly performed by the student that can potentially compromise the quality of care or patient safety. Critical errors resulted in the failure of the OSCE even if the global grade is above the passing threshold. Pilot testing was done on five students, and feedback was gathered from the students and examiners. This was followed by another round of refinement for technical issues, confusing steps, and inconsistencies in the rubric or scoring guide.

The raters for the OSCE were chosen based on their expertise in the field and experience with OSCE assessments. After training the raters and conducting calibration sessions, inter-rater reliability coefficient for more than two raters was determined; Fleiss’s Kappa was calculated by having the raters independently rate each item on the OSCE checklist to ensure a consistent scoring approach. Fleiss’s Kappa was 0.806, p < 0.001, CI [0.669–0.943] at 95%, which is substantial agreement according to Cohen’s Kappa Scale [28].

The OSCE was administered to the students after a briefing session on how to perform the OSCE and multiple practice sessions. The OSCE was conducted in a well-structured and controlled environment, with well-delineated instructions to the students, and adequate training to the SP. The candidate was considered competent in the medical emergency station after the candidate’s global score was determined to be higher than the minimum (70%, pass/fail) threshold, with no critical errors.

### Statistical analysis

The difference between post-test- and pre-test scores was considered the dependent variable in this study. The dependent variable was summarized with mean and standard deviation (SD) and graphically presented using boxplots. Changes in scores were assessed in the overall group and by school year using paired samples t-tests. Pre-test, post-test, and changes in scores by school year for each academic year were also summarized.

For students who completed the course multiple times during successive study years, a linear mixed model with random intercepts was used to assess the impact of repeated course enrollments on the final exam score; the mean change in score and its 95% confidence intervals were estimated.

Chi-square test was used to compare the outcomes (pass/fail rates) of the Year 6 OSCE, with 70% set as the passing threshold. The pass/fail data from 2017 (Year-6 students that underwent the module once) and 2019 (Year-6 students that underwent the module thrice, consecutively in school years 4, 5, and 6) were tabulated. Chi-square test was used to determine whether there was a significant association between the pass/fail rates and the number of times the module was taken.

All data were analyzed using R programming language version 4.0.0. Statistical significance was set at p < 0.05.

## Results

### Number and distribution of student assessments

A total of 846 students participated in the study: 275 once, 483 twice, 87 thrice, and 1 student participated four times. The number and distribution of the students assessed through the school and academic years are shown in Table 1.

**Table 1:** The number and distribution of the assessments by school and academic years

	Academic Year			
	2017	2018	2019	2020
**Year 4 students**	153	137	118	169
**Year 5 students**	99	99	134	128
**Year 6 students**	103	104	140	122
*Total*	355	340	392	419

### Overall change in assessment scores

In total, 1,506 assessments were conducted between 2017 and 2020. Overall, the pre-test and post-test mean scores were 9.4 ± 3.0 and 13.5 ± 1.6, respectively. The mean change in overall scores (4.0 ± 2.7) was statistically significant, p < 0.001.

### Change in assessment scores by school year

**Fig. 2:**
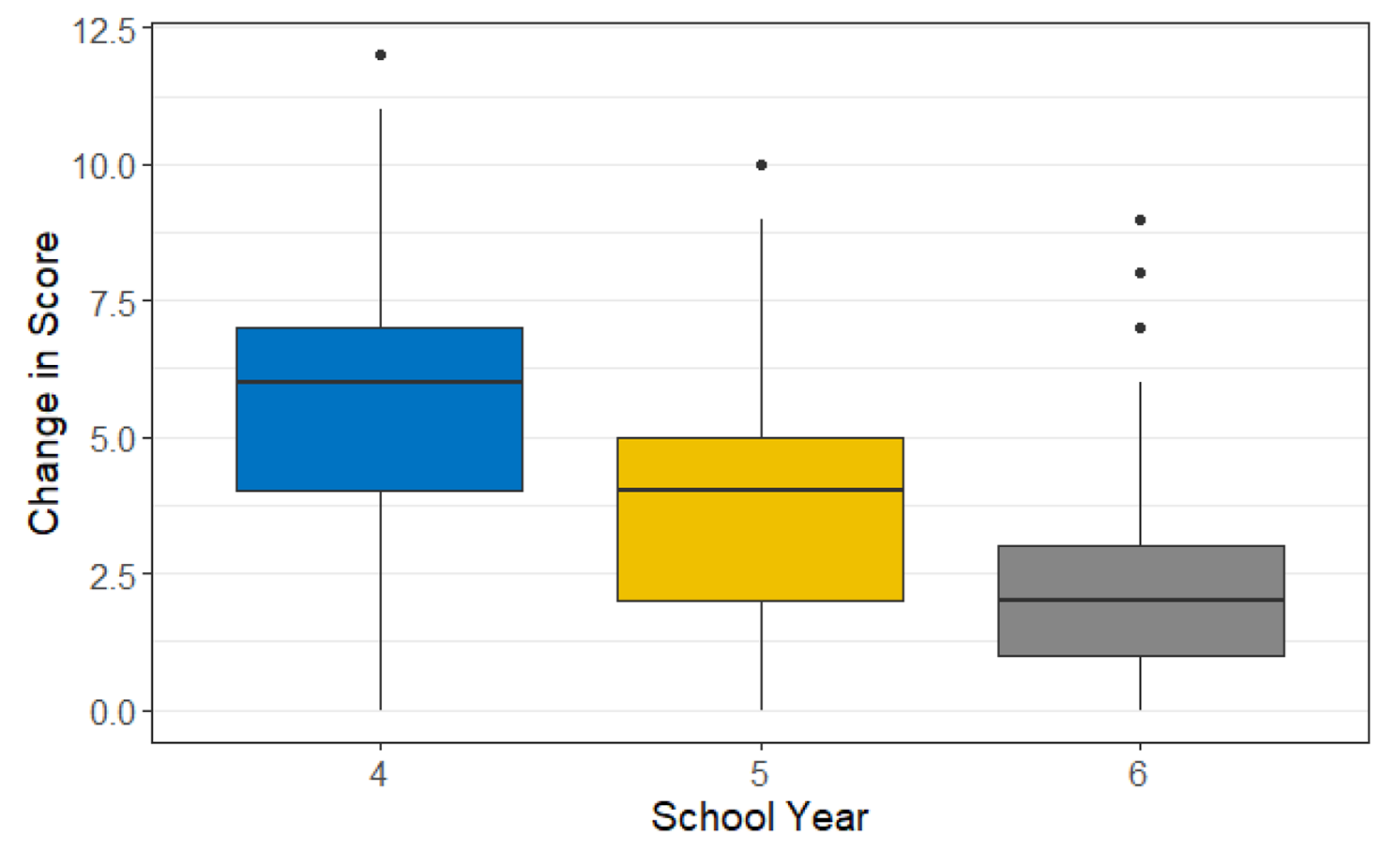
Change in score by school year

On comparing the pre-test to the post-test scores in Years 4, 5, and 6 students, all students showed significant improvement in the post-test scores compared to the pre-test scores (Table 2; Fig. 2).

**Table 2:** Mean (± SD) pre-test, post-test, and difference in scores by school year. P-values are obtained from paired t-tests

	N	Pre-test Score	Post-test Score	Difference (Post-Pre)	P value
**Year 4**	577	6.9 ± 2	12.8 ± 1.7	5.8 ± 2.5	< 0.001
**Year 5**	460	9.8 ± 2.6	13.7 ± 1.6	3.9 ± 2.2	< 0.001
**Year 6**	469	12.2 ± 1.9	14.2 ± 1.1	2.0 ± 1.8	< 0.001

### Change in assessment scores by school year and academic year

**Fig. 3:**
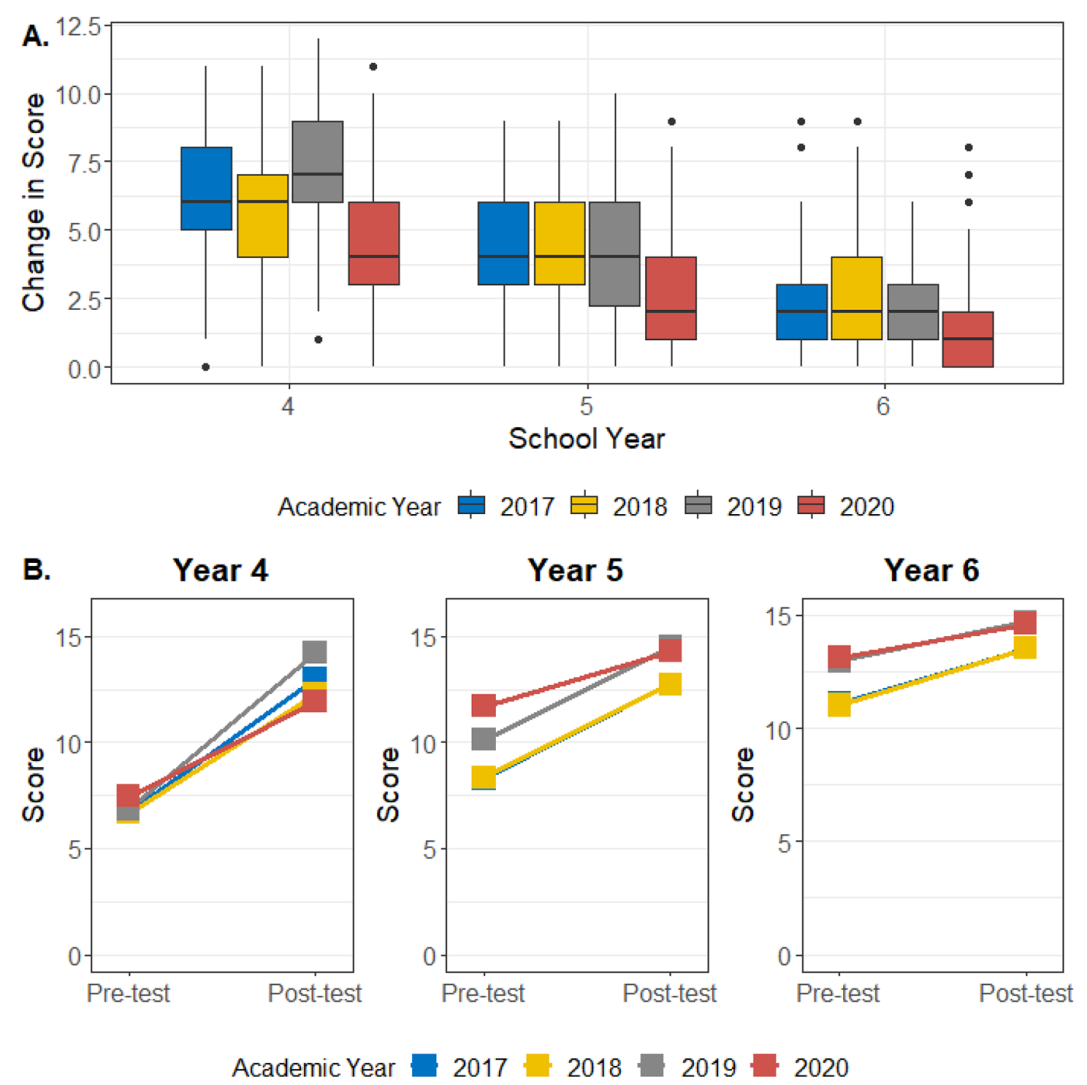
**A)** Change in scores by School year; **B)** Change in scores by academic year with each school year

Year 4 students showed the highest improvement, followed by Year 5 students, and Year 6 students. Table 3; Fig. 3 further break down scores by academic year, where the same trends are observed over time; that is, all students in all three school years showed a positive change in score from pre- to post-test, as reflected by a consistent and statistically significant improvement in the pre-test scores in the successive academic years for every school year.

**Table 3:** Mean (± SD) pre-test, post-test, and change in scores by school year and academic year. P-values from all paired t-tests were < 0.001

School Year	Measure	Academic Year			
		2017	2018	2019	2020
**Year 4**	Pre-test score	6.7 ± 2.0	6.7 ± 2.1	6.8 ± 1.9	7.5 ± 2.1
	Post-test score	13.0 ± 1.1	12.3 ± 1.9	14.2 ± 1.5	11.9 ± 1.5
	*Change in Score*	6.3 ± 2.2	5.6 ± 2.3	7.4 ± 2.5	4.5 ± 2.2
**Year 5**	Pre-test score	8.3 ± 2.1	8.4 ± 2.0	10.1 ± 2.7	11.7 ± 1.9
	Post-test score	12.7 ± 1.6	12.7 ± 1.6	14.5 ± 1.5	14.3 ± 0.8
	*Change in Score*	4.4 ± 1.9	4.4 ± 1.8	4.4 ± 2.4	2.6 ± 2.0
**Year 6**	Pre-test score	11.1 ± 2.0	11.0 ± 2.0	13.0 ± 1.4	13.1 ± 1.4
	Post-test score	13.5 ± 0.8	13.5 ± 0.9	14.7 ± 1.1	14.6 ± 1.0
	*Change in Score*	2.4 ± 1.8	2.5 ± 2.0	1.8 ± 1.5	1.5 ± 1.6

### Longitudinal assessment results: linear mixed model

A total of 1,231 assessments for 571 students were incorporated into the linear mixed model. With each course enrollment, the final exam score increased on average by 0.88 points (95% CI: 0.76–0.99), p < 0.001.

### Association of OSCE pass/fail rate with the frequency of module completion

The Chi-square analysis was statistically significant for the association between the pass/fail rate and the number of times the module was completed, X^2^ = 26.887, *df* = 1, p < 0.001 (Table 4).

**Table 4:** Comparisons of Year-6 OSCE outcomes between students completing the module once and those completing it thrice over three consecutive years

	Year-6 OSCE outcomes in 2017 (module completed once)	Year-6 OSCE outcomes in 2019 (module completed thrice)
PASS	83	140
FAIL	40	12
TOTAL	123	152
*P-value*	< 0.001	

## Discussion

The implementation of our emergency management training module for dental students had several remarkable features. Our module utilized simulation through role-play and mandated it to all dental students on an annual basis. We provided standardized stations for the hands-on training for the equipment and drugs not only as a didactic component but in a hands-on manner. The advantages of this training module were the use of role-play which was not cost-prohibitive to set up, and it allowed the students to engage in many different emergency scenarios. Based on the literature [2, 4, 9, 10, 18, 23–27], the most common medical emergencies were used for the simulation. Furthermore, the role-play allowed the students to exhibit leadership and advance effective communication between team members.

Moreover, our results indicate that this long-term (4-year) program was shown to be reproducible and sustainable. It is an effective model for training undergraduate students, as indicated by the significantly increased improvement in post-test scores compared to pre-test scores every successive academic year for a given school year. This was the fourth consecutive year for this course to run annually, and with a well-established medical emergency system at our institution, this was reflected in the scores in 2020, which were the highest pre-test scores across all academic years.

The medical emergency department at our dental school was established at the same time as the training module. The department policy, procedures, and key performance indicators complimented the training module. The establishment of an effective operating system within the school, the practice of bi-annual mock drills, proper record-keeping, periodic case reviews, and debriefing sessions, all strengthened the training module and contributed to a well-established system within our institution.

The estimates of the prevalence of medical emergencies in the dental office are often calculated based on retrospective surveys conducted among dental practitioners. They are often expressed as events per dentist per year. In such surveys, the prevalence of medical emergency events has been generally reported as between 0.5 and 2 events per dentist per year [6, 7]. Studies have shown that nearly 50–70% of all dental practitioners experience at least one medical emergency event each year, of which more than a quarter could encounter multiple such events in a year [3]. These statistics indicate that a dental practitioner encountering a medical emergency in a dental office is not uncommon despite the low risk of medical emergencies in many individual patients visiting a dental office. Despite its occasional occurrence of a few times a year, the threat of a medical emergency could be assigned a very high priority at the level of dental practitioners due to potentially life-threatening complications in patients and the threat to the professional reputation and satisfaction among dental practitioners [8].

Ideally, all dental practitioners must always be prepared to safely manage medical emergencies in a dental office. Hence, medical emergency management in the dental office is a crucial component of a dentist’s undergraduate training. Before this module, only theoretical lectures were provided to the students; the students were expected to be competent in managing a simulated medical emergency in the final OSCE, which was held annually and mandatory for graduation. In addition, when practicing in the community, the dentists are expected to be able to have proper office protocols for medical emergencies, prepare the equipment required and manage a medical emergency using basic drugs.

Implementation of Continuing Professional Development (CPD) programs that include the topic of management of medical emergencies is one of the prerequisites for attaining adequate preparedness among dental practitioners. The General Dental Council of the United Kingdom recommends regular knowledge and skill training for dental practitioners on the topic of medical emergencies under its CPD program; a total of 10 h per CPD cycle and 2 h per year have been recommended on the topic of medical emergencies to improve patient safety [29].

Training dental students/residents to manage common emergency conditions can improve their preparedness for dental emergencies. Therefore, traditional lecture-based programs are being utilized in most academic institutions. For instance, a 70-hour program distributed over four academic years is used in France [30]. In the US, such programs for dental students are also coupled with the BLS [31] certifications, whereas the programs for dental residents have been integrated with the Advanced Cardiac Life Support certifications [32].

In addition, several novel simulation-based educational courses that use robot patients, simulation models, or cognitive aids are being developed to improve the competency of dental students, residents, or practitioners in emergency management [30–37].

Three of these eight studies included dental students [30, 31, 35]; one study included dental residents [32]; however, four studies focused on dental practitioners, including dentists, dental hygienists, dental assistants, and nurses [33, 34, 36, 37].

The methods used in these studies were predominantly in the form of pre- and post-simulation written examinations and simulation performance scoring. Interestingly, one study used OSCE [32], while another study used a stress response assessment of the participants [35]. Some of these studies used high-fidelity simulation approaches [30, 32], while others have focused on low-cost simulation model approaches [31, 34–37].

Recently, a tele-simulation on medical emergencies in the dental office, which was necessitated by the COVID-19 pandemic, was qualitatively shown by Kishimoto et al. in which they demonstrated the effectiveness of their earlier model [34] in the new tele-simulation setting, thereby revealing additional possibilities of learning through the tele-simulation (blended-learning) mode. However, this may need further demonstration using quantitative methods [36].

Given the short-term improvements seen in almost all of these studies, these simulation models show great promise in the improved management of emergency conditions in a dental office. However, these studies indeed have some limitations; most studies, except the one by Marti et al. [31] included small samples; most studies did not assess their impact on the practice, especially over the long term. In addition, many studies did not have a control group or comparators; only one recent study has compared a simulation approach against other approaches in a randomized controlled trial (RCT), in which the simulation-based approach in the simulation group was found better than the approaches used in the other groups, the educational flipchart group and the control group [37].

As highlighted above, we could find only three recent reports [30, 31, 35] from global academic dental institutions that had used simulation-based approaches for teaching medical emergency management to dental students, when not counting the studies solely dedicated to cardiopulmonary resuscitation. Of these, only the study by Marti et al. [31] from an American teaching dental institution offers a close comparison with our study, as it used comparable methods; the other two used high-fidelity simulator or virtual-reality-based simulator. In the study by Marti et al., the dental students were randomly divided into five groups of 15 students per group; the time spent at each of the five role-play simulation-based stations was 30 min and the overall duration was 3 h. The five stations covered the following themes: “altered consciousness, chest pain, drug-related emergencies, unconsciousness, and respiratory distress.” A 3-year evaluation was conducted on a total of 333 dental students by Marti et al. In contrast, our evaluation included a larger number (846) of dental students over a 4-year period. Our module included six role play simulation-based stations on the themes of syncope/postural hypotension, hypoglycemia, asthma, hyperventilation/epinephrine reaction, allergic reaction, and chest pain besides the four hands-on sessions on emergency medicines, crash cart, monitors and oxygen equipment, and a basic life support refresher session. While the number of topics of our program may be considered more than that of Marti et al., our dental students spent slightly less time (15 min) at each of the 10 stations in our module compared to that of Marti et al. (30 min at each of the 5 stations). Pre-and post-test assessments were conducted by both Marti et al. and us; they used a 25-item student questionnaire, while we used a 16-item test. While we had an additional OSCE component of evaluation, they used additional peer performance assessment checklist. Both studies demonstrated significant improvement in the dental students’ knowledge and performance, which suggests that the role-play simulation-based modules are a valid approach for improving the students’ knowledge and skills (preparedness) related to handling medical emergency management. The impact of the minor differences in the curriculum and structure of the modules may not be large, provided that the module has been designed and developed by the local experts in accordance with local needs. However, a long implementation period of 3–4 years is necessary to document the longitudinal improvement in the students’ preparedness using the role-play simulation-based module.

### Limitations

Despite being a low-cost model with a large sample and four years of successful use, this module/study had some limitations as well, such as being a single-center study from a leading university center with extensive resources. Whether this module can be replicated in other centers, especially those without the support of emergency departments and fewer resources, is yet to be seen. The scalability of this module to other dental professionals, such as dental hygienists and technicians, must also be evaluated before this model can be considered for widespread use. In addition, a 16-item multiple choice test and a one-scenario-based OSCE used in this study might not have adequately and precisely measured the knowledge of the dental students in the management of medical emergencies. Similarly, the role-play simulation used in this study was spearheaded by the instructors who followed a rubric to ensure consistency, but these role-plays were not video-recorded for a later viewing by the dental students. In contrast, a role-play simulation-based study on Saudi Arabian dental students regarding the skills of professionalism used the video recordings, which was found to be a useful strategy by three-fourths of the participating students [38]. Our study, like most previous studies, used a single simulation technique (role-play) to develop competence in medical emergency management among dental students. Studies combining more than one simulation techniques may result in better development of competence among dental students.

Finally, validity of the assessments is a multifaceted concept. Additionally, determining competency in the medical field is complex and challenging. The validity of the test and OSCE used here was based on content validity, which is a traditional approach. Since the module now is well-established and integral part of our curriculum, we believe it would be best if a more contemporary approach to validity of the OSCE is used, namely the Kane’s Validity Framework, which is gaining interest in the healthcare field [39].

## Conclusions

The successful evaluation of this role-play simulation-based clinical training module supports integrating this module on management of medical emergencies into the dental education. It is advisable to use this module as an annual mandate for all dental students in their clinical years. Our study suggests that academic dental institutions can effectively utilize simulation and hands-on instruction methodologies to teach medical emergency management to dental students and raise their competency levels in preparedness, prevention, and management of any unexpected medical emergency during dental treatment.

## Data Availability

Datasets are available upon request from the author; Maisa O. Al-Sebaei, moalsebaei@kau.edu.sa.

## List of abbreviations

BLS: Basic Life Support
COVID-19: Coronavirus Disease 2019
CPD: Continued Professional Development
KAUFD: King Abdulaziz University Faculty of Dentistry
OSCE: Objective Structured Clinical Exam
RCT: Randomized Controlled Trial
SD: Standard Deviation
SP: Standardized Patient
